# Image‐free robotic‐assisted total knee arthroplasty is associated with joint line distalization and improves mid‐flexion instability: A prospective cohort study

**DOI:** 10.1002/jeo2.70239

**Published:** 2025-04-13

**Authors:** Keisuke Maeda, Tomoharu Mochizuki, Shigeru Takagi, Go Omori, Noriaki Yamamoto, Koichi Kobayashi, Hiroyuki Kawashima

**Affiliations:** ^1^ Division of Orthopedic Surgery Niigata University Medical and Dental Hospital Niigata Japan; ^2^ Department of Orthopedic Surgery Niigata Rehabilitation Hospital Niigata Japan; ^3^ Department of Health and Sports Niigata University of Health and Welfare Niigata Japan; ^4^ School of Health Sciences, Faculty of Medicine Niigata University Niigata Japan

**Keywords:** joint line, mid‐flexion instability, robotic‐assisted, total knee arthroplasty

## Abstract

**Purpose:**

Total knee arthroplasty (TKA) has demonstrated long‐term durability, with a significant reduction in revisions due to polyethylene wear and component loosening. However, mid‐flexion instability (MFI) is a key factor in early TKA revisions, affecting patient satisfaction and implant longevity. Recent advancements in robotic‐assisted TKA (raTKA) provide precise joint line (JL) restoration and component positioning, potentially reducing MFI. This prospective study evaluated the impact of image‐free raTKA on MFI and JL restoration.

**Methods:**

This prospective cohort study included 59 knees undergoing primary TKA using the image‐free robotic systems NAVIO® and CORI® and the JOURNEY II® Bi‐Cruciate Stabilized knee system. Intraoperative component gap (CG) measurements at 0°, 30°, 60° and 105° of flexion were taken, and JL changes were assessed pre‐ and post‐operatively using computed tomography (CT)‐based three‐dimensional (3D) models with the 3D‐3D matching technique. The distal femoral JL was quantified.

**Results:**

Both the medial and lateral CG at 30° and 60° were significantly smaller compared to those at 0° and 105°. Post‐operative JL showed distalization of 1.5 mm at the medial femur and 2.0 mm at the lateral femur compared to preoperative JL.

**Conclusions:**

This study is the first to assess JL restoration in raTKA using CT‐based bone landmarks, offering precise insights. Image‐free raTKA facilitates precise JL restoration, optimizing knee kinematics and enhancing stability. These findings suggest that this technique contributes to improved post‐operative joint function and greater patient satisfaction.

**Level of Evidence:**

Level II, prospective cohort study.

**Abbreviations**
3D: three‐dimensional
CG: component gap
CT: computed tomography
HKA: hip–knee–ankle
JL: joint line
MA: mechanical axis
MCL: medial collateral ligament
MFI: mid‐flexion instability
raTKA: robotic‐assisted TKA
SD: standard deviation
SEA: surgical epicondylar axis
TKA: total knee arthroplasty

## INTRODUCTION

In recent years, total knee arthroplasty (TKA) has demonstrated exceptional long‐term durability, with significant reductions in revisions attributable to polyethylene wear and component loosening [4, 19, 28]. However, joint instability has emerged as a significant issue, contributing to revisions in the short‐ to mid‐term period [20, 22]. Notably, it has been reported that approximately 3% of TKA cases require revision within 2 years post‐operatively, with instability accounting for 20%–30% of these revisions [12, 36]. Furthermore, even in cases where instability does not necessitate revision, post‐operative joint instability may contribute to suboptimal joint function and diminished patient satisfaction [3, 10, 34], particularly within the extension to mid‐flexion ranges, where stability is crucial for daily activities such as walking. The concept of mid‐flexion instability (MFI), initially introduced by Martin and Whiteside [24] through an experimental study, has garnered increasing attention, with recent clinical studies further highlighting its relevance [14, 26, 29]. The aetiology of MFI has been attributed to various factors [9, 11, 14, 24, 26, 29, 33], with previous cadaver studies indicating that the joint line (JL) elevation can result in MFI after TKA [23, 24]. Therefore, accurately restoring the JL during TKA is considered critically important.

Since the 1990s, computer‐assisted surgery gradually become more widely adopted in TKA, demonstrating more accurate alignment outcomes compared to conventional techniques [13, 25]. Recently, advancements in robotics have introduced a next‐generation image‐free handheld robotic sculpting system that does not require preoperative imaging, enabling surgeons to plan implant positioning intraoperatively with six degrees of freedom. This image‐free robotic‐assisted TKA (raTKA) not only facilitates more precise osteotomies than traditional navigation systems [7] but also allows for intraoperative adjustments to bony alignment to optimize soft tissue balancing [5, 27]. The intraoperative morphing process also generates a model that accurately restores the JL, including the cartilage. Thus, compared to conventional TKA, image‐free raTKA offers the potential for achieving the desired bony alignment through highly precise osteotomies, accurate restoration of the JL, including the cartilage, and optimization of soft tissue balance, which may subsequently improve laxity in the mid‐flexion range—a condition influenced by multiple factors. However, no studies have examined the relationship between raTKA, MFI and JL. It has been hypothesized that image‐free raTKA would stabilize laxity in the mid‐flexion range and conducted a prospective study to evaluate this hypothesis.

## MATERIALS AND METHODS

The Ethical Review Board of Niigata University approved this prospective observational study (IRB number: 2020‐0448).

**Figure 1:**
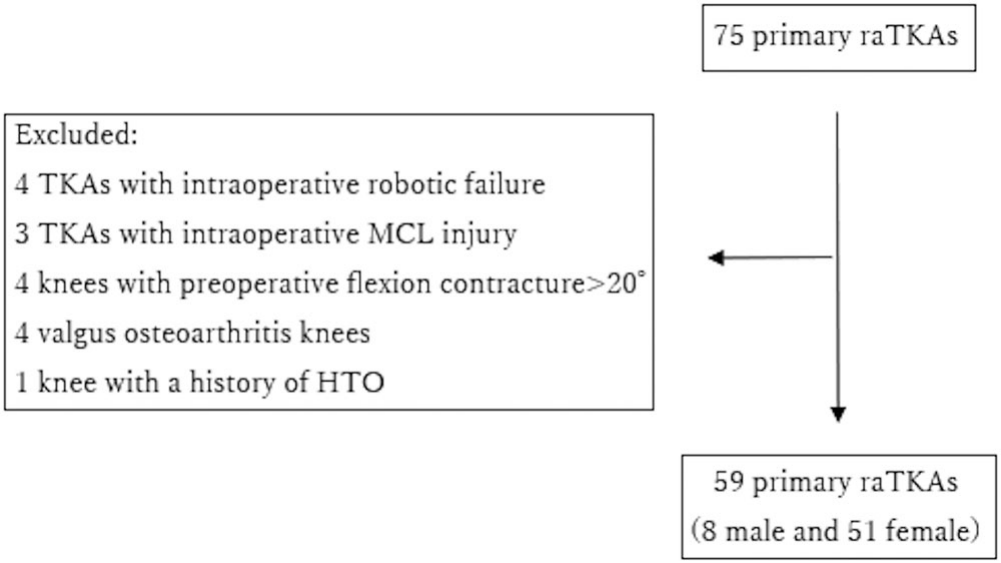
Flowchart of patients undergoing raTKA. HTO, high tibial osteotomy; MCL, medial collateral ligament; raTKA, robotic‐assisted TKA; TKA, total knee arthroplasty.

From September 2021 to March 2024, a total of 75 primary TKA were performed. Inclusion criteria: patients undergoing primary raTKA with the NAVIO® or CORI® systems using the JOURNEY II® Bi‐Cruciate Stabilized knee system. Exclusion criteria: patients requiring manual conversion, MCL repair, preoperative flexion contracture >20°, valgus osteoarthritis or prior high tibial osteotomy. Missing data were excluded from the analysis. Ultimately, 59 knees (7 NAVIO® knees and 52 CORI® knees) met the inclusion criteria for the study. All cases involved patients with osteoarthritis, with no instances of osteonecrosis or rheumatoid arthritis. The cohort consisted of 8 male and 51 female patients (Figure 1). The patients' demographic data, including age, height, weight, body mass index and preoperative hip–knee–ankle (HKA) angle, were summarized in Table 1.

**Table 1:** Demographic data.

	Mean ± SD	Median (range)
Age, years	73.5 ± 6.5	74.0 (60.0–87.0)
Body height, cm	153.8 ± 7.6	153.2 (133.0–173.0)
Body weight, kg	61.0 ± 10.8	57.5 (44.0–91.0)
Body mass index, kg/m^2^	25.9 ± 3.6	24.7 (19.5–34.3)
Pre‐flexion angle, °	124.5 ± 15.1	130.0 (80.0–155.0)
Pre‐extension angle, °	−5.3 ± 4.7	−5.0 (−20.0–0.0)
Pre‐HKA, °	190.6 ± 6.1	190.0 (181.0–212.0)

Abbreviations: HKA, hip–knee–ankle angle; SD, standard deviation.

### Surgical procedure

**Figure 2:**
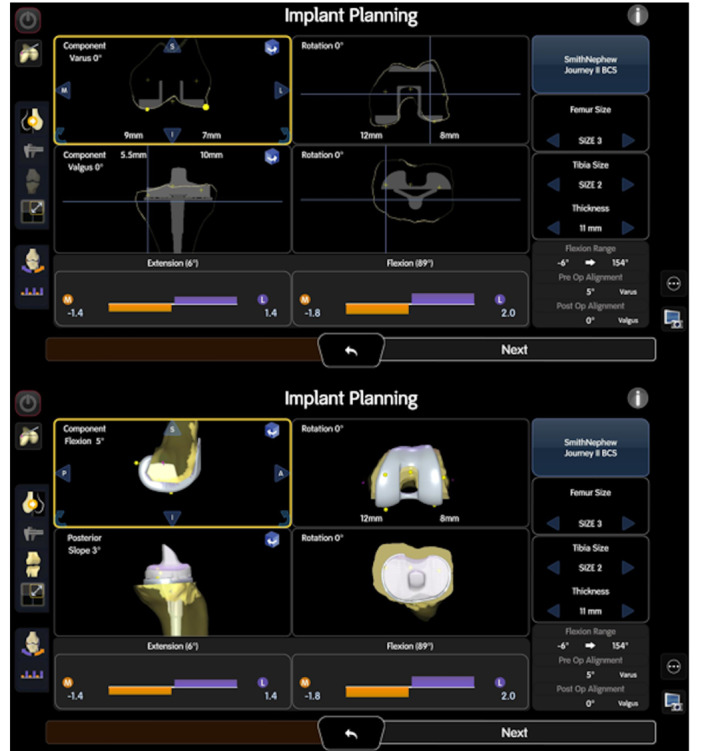
Navio® and CORI®. Implant planning according to intraoperative soft tissue balance and three‐dimensional model built with image‐free smart mapping.

The Navio® and CORI® surgical systems, both handheld, image‐free and semi‐active robotic platforms, were employed in this study. These systems facilitate real‐time planning and personalized surgery through image‐free smart mapping, enabling the construction of three‐dimensional (3D) models of the joint intraoperatively without the need for preoperative computed tomography (CT) or magnetic resonance imaging scans (Figure 2). The surgical procedures associated with both systems are analogous, offering features such as image‐free mapping of bone geometry, intraoperative planning, gap assessment, and confirmation of alignment and knee balance. For preoperative planning, the 3D software (JIGEN®; LEXI, Inc.) was utilized to determine the size and default positioning of the femoral and tibial components. The default femoral component position was set to 0° relative to the mechanical axis (MA) in coronal alignment, aiming to restore the JL of either the medial or lateral joint surface and to ensure that neither the medial nor lateral JL was proximalized during bone cutting, 3° flexion relative to the MA in sagittal alignment, and 0° relative to the surgical epicondylar axis (SEA) in rotational alignment. Similarly, the default tibial component position was established at 0° relative to the MA in coronal alignment, aiming to restore the JL of the lateral joint surface, with a 3° posterior inclination to the MA in sagittal alignment, and 0° relative to the Akagi line [2] or using the range of motion technique for rotational alignment. Intraoperatively, the positions of the femoral, rather than the tibial components, were fine‐tuned while considering soft tissue balance and total lower limb alignment. The femoral component adjustments ranged from 0° to 3° varus alignment to the MA in the coronal plane, 0° to 6° flexion alignment to the MA in the sagittal plane, and ±3° relative to the SEA in rotational alignment. The tibial component positions remained fixed at 0° to the MA in the coronal plane and a 3° posterior inclination to the MA in the sagittal plane. The tibial rotational alignment was determined as described above by the Akagi line or the range of motion technique. The target gap was set to achieve medial and lateral gaps that were either equal or slightly looser on the lateral side, and extension and flexion gaps that were either equal or slightly looser in flexion. These adjustments were fine‐tuned within the previously mentioned range of femoral component positioning angles. Consequently, the overall lower limb alignment was finalized within a 0–3° varus alignment relative to the MA in coronal alignment. Patella resurfacing was not performed in any case.

### Intraoperative component gap (CG) measurement

**Figure 3:**
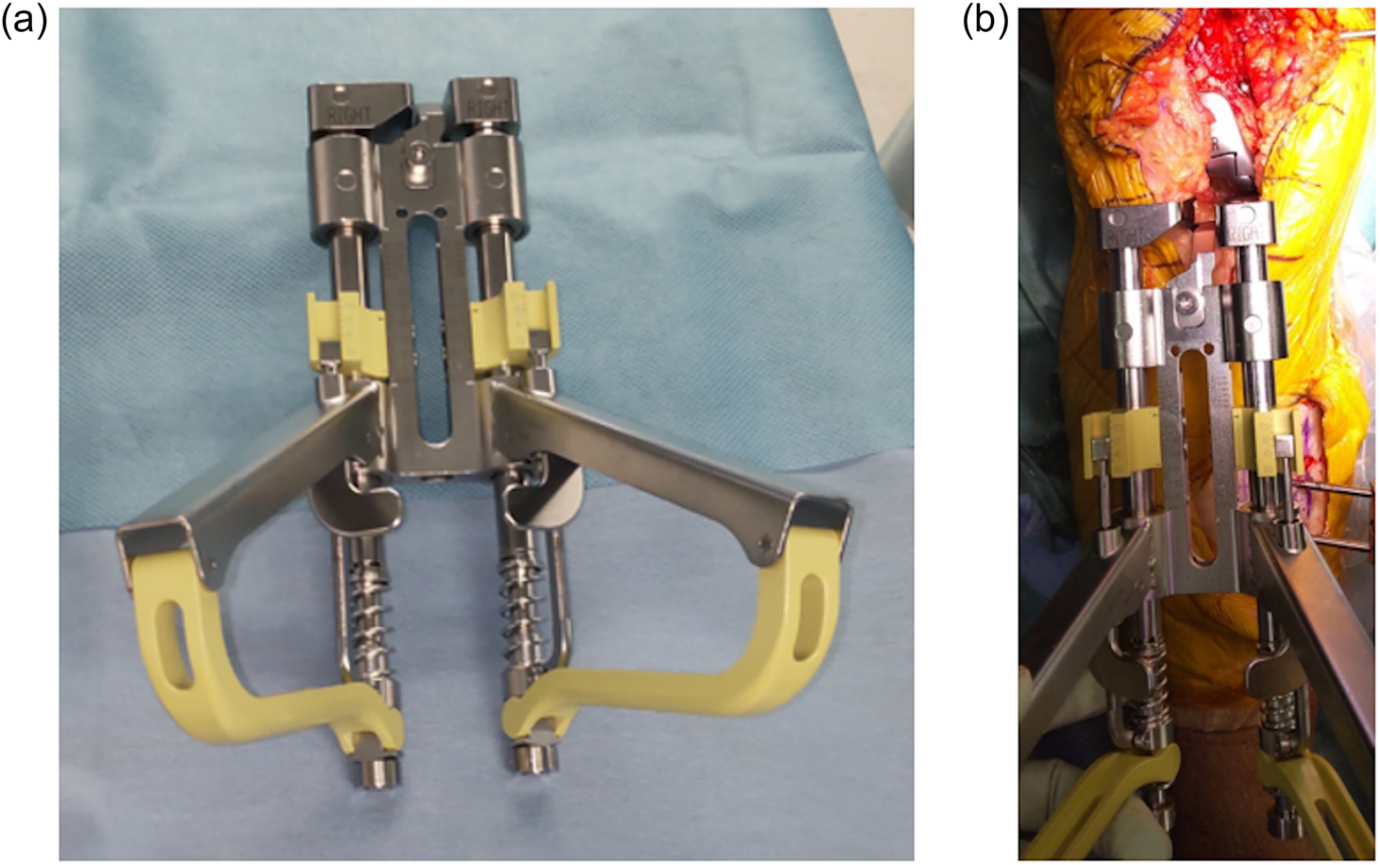
Compartment‐specific ligament tensioner. (a) The ligament tensor can independently evaluate the component gap in the medial and lateral compartments. (b) The picture of the intraoperative measurement of the component gap.

After resecting the femur and tibia, a femoral trial implant was positioned, and the CG was measured using a compartment‐specific ligament tensioner (Figure 3) [15]. A distraction force of 80 N was applied to both the medial and lateral compartments at knee flexion angles of 0°, 30°, 60° and 105°. The patellofemoral joint was reduced during these gap measurements to ensure accurate assessment. The Journey II implant system recommends measuring flexion bone gaps at 105°, aligning with its five‐plane bone resection protocol. However, CG at 90° was not recorded in this study, limiting direct comparison with prior literature.

### JL evaluation

Preoperative CT scans of the femur and tibia were obtained for each subject, with images captured at 1‐mm intervals. These scans were used to evaluate the change in the JL through 3D evaluation and to measure bony resection amounts based on mechanical alignment in the preoperative plan, serving as a reference to ensure that the intraoperative plan did not deviate significantly. They were further processed to construct 3D models using ZedView® (LEXI Inc.) and to establish anatomical coordinate systems, following the definitions reported by Sato et al. [31]. Additionally, the most distal points of the medial and lateral femoral condyles were identified for subsequent analysis.

The preoperative JL was assessed by analyzing the coordinates of the anatomical reference points in the 3D bone models. For the femur, the preoperative distal JL was defined by the *z*‐axis coordinate of the distal‐most points on the medial and lateral femoral condyles.

**Figure 4:**
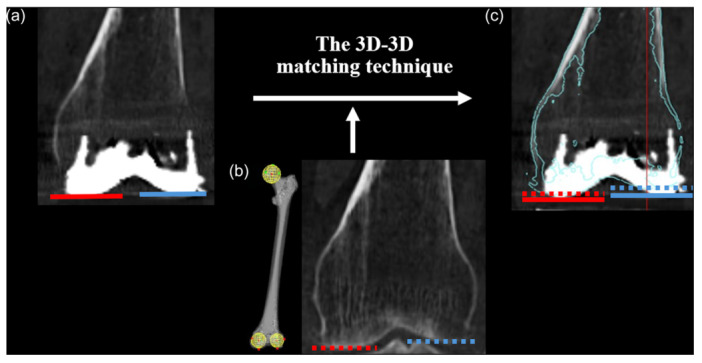
Joint line (JL) evaluation. (a) Post‐operative JL: a red solid line represents the medial JL, and a blue solid line represents the lateral JL. (b) Preoperative JL and three‐dimensional (3D) bone model with anatomical coordinate systems: a red dot line represents the medial JL, and a blue dot line represents the lateral JL. (c) JL Change assessment: Changes in the JL are evaluated using the 3D‐3D matching technique.

The 3D‐3D matching technique (JIGEN®; LEXI, Inc.) was utilized, employing an automated shape‐matching algorithm to align preoperative and post‐operative CT images to evaluate the change in the JL. This method allowed for the determination of the post‐operative JL using the same anatomical coordinate systems established in the preoperative 3D model. For the femur, the post‐operative distal JL was defined by the *z*‐axis coordinate of the distal‐most points on the medial and lateral condyles of the femoral component (Figure 4). The change in the distal JL of the femur was quantified by subtracting the *z*‐axis coordinates of the medial and lateral post‐operative distal JL from the corresponding *z*‐axis coordinates of the medial and lateral preoperative distal JL (Figure 4).

### Statistical analyses

Normality of data was confirmed using the Shapiro‐Wilk test. One‐way analysis of variance with Tukey's post hoc test was applied for normally distributed data, while the Kruskal–Wallis test with Bonferroni correction was used for non‐normal data. Statistical significance was determined at *p* < 0.05. All statistical analyses were performed using SPSS software (version 27; SPSS Inc.).

A power analysis (Cohen's *d* = 0.5, *α* = 0.05, power = 75%) indicated a minimum sample size of 57 patients, confirming that our cohort (*n* = 59) meets statistical requirements.

## RESULTS

### Intraoperative CG

**Figure 5:**
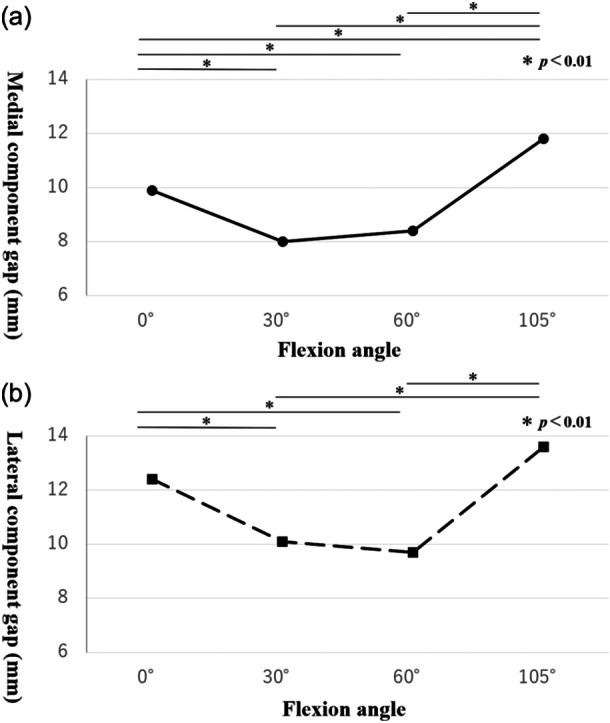
Intraoperative component gap (CG). (a) Medial CG at knee flexion angles of 0°, 30°, 60° and 105°. (b) Lateral CG at knee flexion angles of 0°, 30°, 60° and 105°.

The intraoperative medial and lateral CGs are presented in Table 2. The medial CG (mean ± standard deviation [SD]) at knee flexion angles of 0°, 30°, 60° and 105° was 9.9 ± 1.4, 8.0 ± 1.8, 8.4 ± 2.1 and 11.8 ± 2.4 mm, respectively. The lateral CG (mean ± SD) at the same flexion angles was 12.4 ± 1.9, 10.1 ± 2.3, 9.7 ± 2.5 and 13.6 ± 2.5 mm, respectively. Both the medial and lateral CGs at knee flexion angles of 30° and 60° were significantly smaller compared to those at 0° and 105° (*p *< 0.01) (Figure 5).

**Table 2:** Medial and lateral CG.

	Mean ± SD	95% CI
Medial CG		
CG 0°	9.9 ± 1.4	9.6–10.3
CG 30°	8.0 ± 1.8	7.5–8.4
CG 60°	8.4 ± 2.1	7.9–8.9
CG 105°	11.8 ± 2.4	11.2–12.4
Lateral CG		
CG 0°	12.4 ± 1.9	11.9–13.0
CG 30°	10.1 ± 2.3	9.6–10.7
CG 60°	9.7 ± 2.5	9.1–10.4
CG 105°	13.6 ± 2.5	13.0–14.2

Abbreviations: 95% CI, 95% confidence interval; CG, component gap; SD, standard deviation.

### Post‐operative change in JL

The preoperative and post‐operative JL changes are presented in Table 3. Compared to the preoperative JL, the post‐operative JL showed a distalization of 1.5 ± 1.9 mm at the distal medial femur and 2.0 ± 1.8 mm at the distal lateral femur.

**Table 3:** Post‐operative change in JL.

	Mean ± SD	95% CI
Change in JL height		
MFC JL height	−1.5 ± 1.9	−2.0 to −1.0
LFC JL height	−2.0 ± 1.8	−2.4 to −1.5

*Note*: Positive values represent JL proximalization. Negative values represent JL distalization.

Abbreviations: 95% CI, 95% confidence interval; JL, joint line; LFC, lateral femoral condyle; MFC, medial femoral condyle; SD, standard deviation.

## DISCUSSION

The key findings of this study are that in image‐free raTKA using the JOURNEY II® system, (1) the post‐operative JL demonstrated distalization compared to the preoperative JL when evaluated using CT‐based bone landmarks and (2) the CG in the mid‐flexion range (30° and 60°) was significantly decreased compared to the CG in full extension (0°) and deep flexion (105°).

Several studies have demonstrated that raTKA results in better preservation of the JL compared to conventional TKA [1, 8, 21, 30, 35]; however, all these evaluations were based on radiographic analysis. This study uniquely applies CT‐based 3D matching to quantify JL changes in raTKA, improving accuracy over radiographic methods. We used pre‐ and post‐operative CT scans to conduct a 3D evaluation within the same coordinate system, offering a more precise assessment of JL changes than those provided by earlier radiographic studies. Additionally, earlier studies included cases with severe flexion contractures (>30°) [1, 21, 35], which may have necessitated additional distal femoral resection to achieve an adequate extension gap, potentially resulting in proximalization of the JL. In contrast, this study included only cases with a flexion contracture of 20° or less, ensuring a more accurate evaluation of JL restoration.

In this study, the post‐operative JL was distalized by 1.5 mm at the distal medial femoral condyle and 2.0 mm at the distal lateral femoral condyle compared to the preoperative JL. The observed JL distalization (medial: 1.5 mm, lateral: 2.0 mm) exceeds the typical range reported in prior literature (Cozzarelli et al. [8] 0.04 mm, Agrawal et al. [1] 0.334 mm). However, further studies are required to determine if this degree of distalization translates to measurable clinical benefits. For instance, Popat et al. [30] found that all patients undergoing raTKA had JL changes of less than 5 mm. In contrast, approximately 30% of patients undergoing conventional TKA exhibited a JL change greater than 5 mm. Cozzarelli et al. [8] reported that the post‐operative JL was proximalized by 0.5 mm in conventional TKA compared to just 0.04 mm in raTKA (*p* = 0.030). Similarly, Liow et al. [21] reported a JL change of 3.5 mm in conventional TKA versus 1.9 mm in raTKA (*p *= 0.01). Agrawal et al. [1] also demonstrated that the post‐operative JL change was 2.304 mm in conventional TKA compared to 0.334 mm in raTKA (*p* < 0.001). These findings collectively suggest that raTKA offers a more accurate restoration of the JL than conventional TKA. In this study, the use of pre‐ and post‐operative CT for 3D‐3D matching allowed evaluation within the same coordinate system, which may have resulted in greater distalization of the JL compared to previous studies using x‐rays.

In image‐free raTKA, the intraoperative planning accounts for the joint's shape, including the cartilage. If the resection of the distal femoral condyle is set to match the implant thickness (JOURNEY II®: 9.5 mm medial, 7 mm lateral), the post‐operative JL is distalized by the thickness of the remaining cartilage compared to the preoperative JL when evaluated using CT‐based bone landmarks. As a result, the JL was generally distalized by the amount of remaining cartilage in our study. Consistent with these findings, a previous study using image‐free raTKA [30] reported that the JL was proximalized by 0.91 mm in conventional TKA and distalized by 0.38 mm in raTKA. These findings suggest that image‐free raTKA, by restoring the JL, including the remaining cartilage, results in a distalized JL when evaluated based on bony landmarks via CT. Based on the results of this study, accurately restoring the intraoperative JL including cartilage may lead to favourable outcomes in image‐free raTKA. This approach may contribute to improved stability in the mid‐flexion range.

In this study, the CG in the mid‐flexion range (30° and 60°) was significantly decreased compared to the CG in full extension (0°) and deep flexion (105°) in both medial and lateral compartments. While CG reduction suggests improved MFI, the absence of post‐operative clinical scores (e.g., WOMAC and OKS) limits definitive conclusions. Future research should correlate intraoperative CG changes with functional outcomes.

Several studies have explored the relationship between MFI and femoral component design [6, 32], but a consensus has yet to be reached. For instance, Stoddard et al. [32] found no significant difference in MFI between single‐radius and multi‐radius femoral components. In contrast, Clary et al. [6] reported that mid‐flexion stability was improved with a gradually changing radius femoral component.

**Figure 6:**
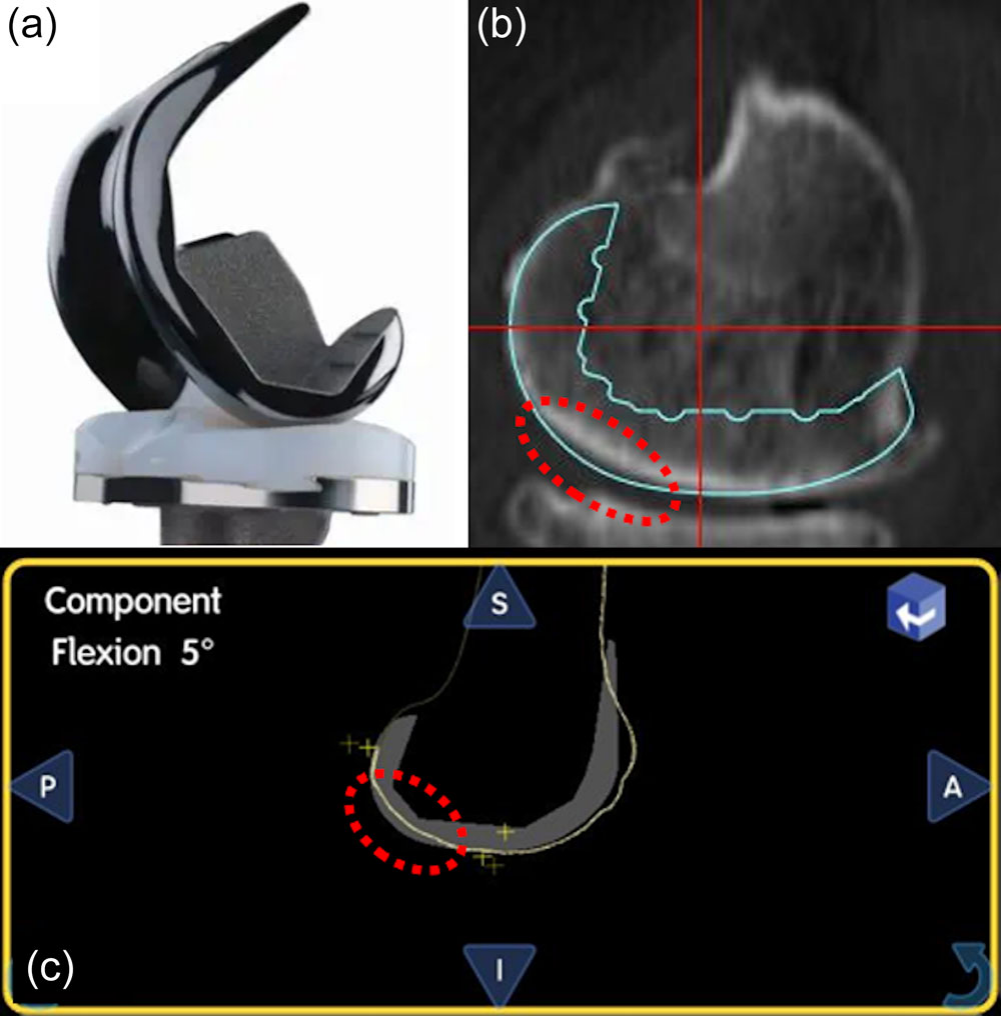
Implant design and planning. (a) JourneyⅡ® implant. (b) Preoperative planning. (c) Intraoperative planning. The red dotted circles indicate implant overstuffing in the mid‐flexion area.

The JOURNEY II® implant used in this study features a gradually changing radius with a J‐curve design. This design results in a longer distance from the radius centre of curvature at 0° flexion to the articular surface in the mid‐flexion range than at 0° and 90° flexion. During both preoperative and intraoperative planning, the implant often protrudes slightly in the mid‐flexion range (Figure 6), which suggests that the component design may contribute to stabilizing the CG in this range. However, previous studies on conventional TKA using the JOURNEY II® system [16, 18] have shown that while mid‐flexion range laxity was more stable than other components, it was still slightly looser than extension. Consistent with our findings, Inui et al. [17] reported that TKA using image‐free navigation with the JOURNEY II® system demonstrated reduced laxity in the mid‐flexion range compared to 10° and 90° flexion. Therefore, the results of the present study may be attributed to the combined effects of accurate JL restoration, including cartilage and the specific component design.

This study has some limitations. First, the relatively small sample size (*n* = 59) may impact statistical power; future multicenter studies should validate these findings. Second, the study did not examine the relationship between CG and clinical outcomes. Nevertheless, previous reports have indicated that instability in the mid‐flexion range can negatively impact clinical evaluations.

## CONCLUSION

This study uniquely applies CT‐based 3D matching to quantify JL changes in raTKA, improving accuracy over radiographic methods and providing precise insights into the impact of this technique. Image‐free raTKA accounts for joint shape, including cartilage, and helps maintain accurate JL alignment, which enhances stability in the mid‐flexion range. These findings suggest that this technique contributes to better post‐operative joint function and increased patient satisfaction.

## AUTHOR CONTRIBUTIONS

Keisuke Maeda and Tomoharu Mochizuki conceived the study. Keisuke Maeda and Tomoharu Mochizuki designed the study. Koichi Kobayashi made the analyzing software. Keisuke Maeda, Tomoharu Mochizuki and Go Omori collected. Keisuke Maeda analyzed the data, and Keisuke Maeda and Tomoharu Mochizuki drafted the initial manuscript. All authors gave critical review and advice on the study design and interpretation. All authors contributed to reviewing and revising the manuscript and agreed on the final draft.

## CONFLICT OF INTEREST STATEMENT

The authors declare no conflicts of interest.

## ETHICS STATEMENT

All procedures performed in studies involving human participants performed in this study were in accordance with the ethical standards of the institutional and/or national research committee and with the 1964 Helsinki Declaration and its later amendments or comparable ethical standards. Informed consents were obtained from all the patients.

## Data Availability

The data sets generated and analyzed during the current study are available from the corresponding author on reasonable request.
